# Feedback in the clinical setting

**DOI:** 10.1186/s12909-020-02280-5

**Published:** 2020-12-03

**Authors:** Annette Burgess, Christie van Diggele, Chris Roberts, Craig Mellis

**Affiliations:** 1 The University of Sydney, Faculty of Medicine and Health, Sydney Medical School - Education Office, The University of Sydney, Edward Ford Building A27, Sydney, NSW 2006 Australia; 2 The University of Sydney, Faculty of Medicine and Health, Sydney Health Professional Education Research Network, The University of Sydney, Sydney, Australia; 3 The University of Sydney, Faculty of Medicine and Health, The University of Sydney, Sydney, Australia; 4 The University of Sydney, Faculty of Medicine and Health, Sydney Medical School, Central Clinical School, The University of Sydney, Sydney, Australia

**Keywords:** Feedback, Peer teaching, Clinical teaching, Student peer-to-peer feedback

## Abstract

Provision of feedback forms an integral part of the learning process. Receipt of feedback enriches the learning experience, and helps to narrow the gap between actual and desired performance. Effective feedback helps to reinforce good practice, motivating the learner towards the desired outcome. However, a common complaint from learners is that the receipt of feedback is infrequent and inadequate. This paper briefly explores the role of feedback within the learning process, the barriers to the feedback process, and practical guidelines for facilitating feedback.

## Background

**Fig. 1:**
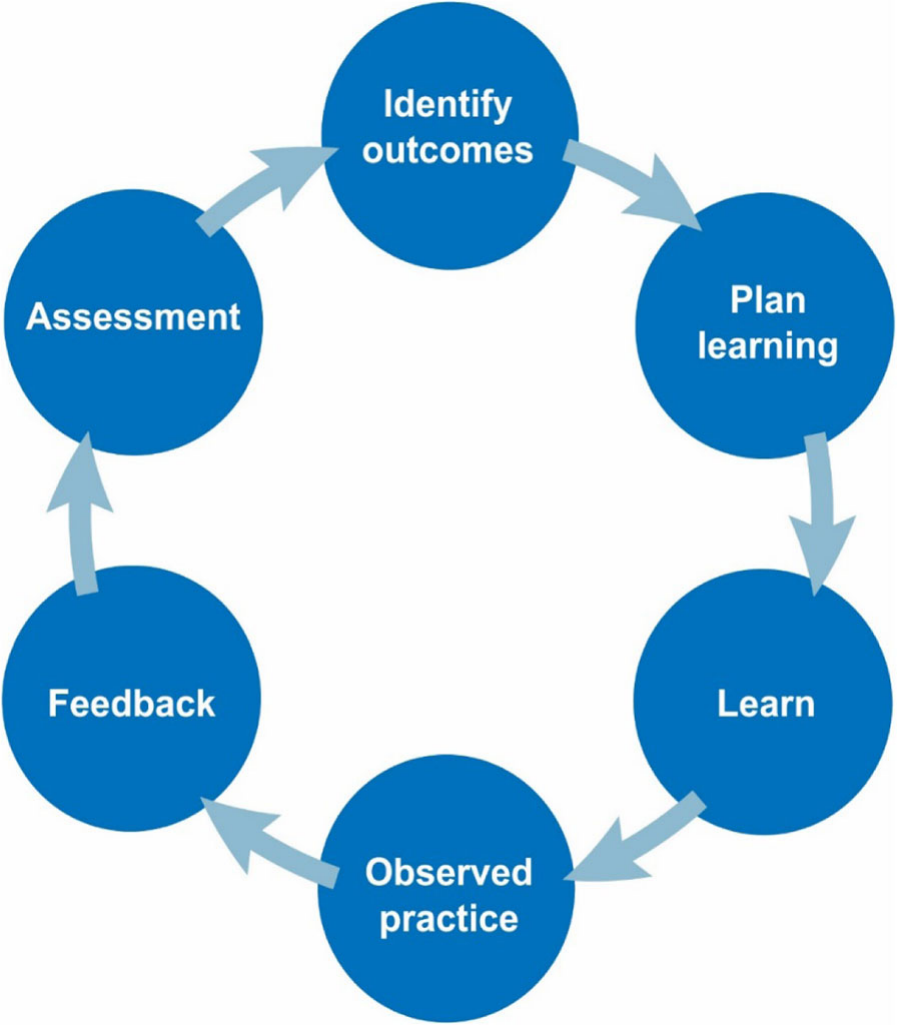
The learning cycle during clinical placements

Within health professional education, feedback has been described as “*Specific information about the comparison between a trainee’s observed performance and a standard, given with the intent to improve the trainee’s performance”* [1]. Feedback is one of the most important forms of interactions between the ‘teacher’ and the ‘learner’. However, it has been widely reported that medical and other health professional students are rarely directly observed and given feedback during their clinical placements [2]. Accordingly, there has been increased interest in the facilitation of feedback [2]. Provision of feedback forms an integral part of the learning process (Fig. 1) [2], helping to narrow the gap between actual and desired performance. The feedback process engages the learner with information about the quality of their performance, and leads to improvements in learning strategies. Feedback supports learners’ effective decision making, and helps to improve learning outcomes. It serves as a powerful tool to provide the learner with judgements on their performance, assisting in their educational progress. However, health professional educators, students and peers can find it difficult to learn from one and other through feedback practices [3]. Feedback practices are often unsustainable, and de-motivating for students [3, 4]. The ability to assess and provide feedback is a learnt skill, requiring an appropriate level of training.

This paper briefly explores the role of feedback within the learning process, barriers to the feedback process, and practical guidelines for facilitating feedback.

### Purpose of feedback

Feedback acts as a continuing part of the instructional process that supports and enhances learning [5]. It is part of an ongoing unit of instruction and assessment, rather than a separate educational entity [6]. A core component of formative assessment [7], feedback promotes learning in three ways [5]:

- Informs the student of their progress
- Informs the student regarding observed learning needs for improvement
- Motivates the student to engage in appropriate learning activities

### Creating a supportive environment for feedback

Requirements for sustainable and meaningful feedback shifts the focus from the provision of feedback to the design of the learning environment that promotes facilitation of feedback [3]. Rather than facilitating individual acts of information provision and reception, feedback should be viewed as the promotion of active learning. Teachers are responsible for fostering interactions between students and their peers, and students and staff. Learning environments should be created where students see themselves as agents of their own change, fostering self-regulation and driving their own learning. Fostering high levels of student engagement helps to develop the identity of students as proactive ‘learners’, who seek feedback and reflect on their own performance.

### Barriers to the feedback process

The process of feedback requires interaction and direction, and should be viewed as essential to clinical education. In the absence of feedback, the uncertainty of a new clinical environment for a learner is intensified. There may be a number of barriers to the feedback process, including:

- *Lack of direct observation of tasks*. Feedback has the greatest impact on students’ behaviour when it is provided based on direct observation of a specific task [2]. In the busy clinical setting, direct observation is often lacking.
- *The desire to avoid upsetting students with honest and critical feedback* [2]*.* Feedback can be more difficult to provide when the learner’s performance is below par, and may be disappointing to the learner. The provision of such feedback requires an understanding of the process, and skill. Although there may be a desire to avoid upsetting a learner, this can result in “vanishing feedback” [8], where meaningful feedback is avoided.
- *Lack of external feedback.* Without external feedback, students may generate their own feedback - but, self-assessment is often wrong [4]. High performers tend to underestimate their own performance, and lower performers tend to overestimate [9].

### Learner reception of feedback

Similar to giving feedback, receiving feedback is not a passive, simple act. It entails honest self reflection and commitment to practice and improvement of clinical skills. Learners are not always prepared for receiving, and more importantly, accepting feedback. Additionally, there may be contextual and relational aspects regarding the feedback [10]. Clearly, acceptance and effectiveness of the feedback may be dependent upon the perceived credibility of the provider [10]. The learner is more accepting of the feedback if they perceive the provider to have a good understanding of the curriculum, and the learning objectives.

### Student peer-to-peer feedback

The practice of providing feedback to peers is perceived by students as beneficial to development of knowledge, skills, and professional attributes [11]. Provision of feedback from peers can foster high levels of responsibility in students [11, 12], and some students report metacognitive gains [11–13]. However, unsurprisingly, there are real concerns regarding the honesty and accuracy of peer feedback [11–15]. The inability of students to provide constructive feedback to peers has been attributed to both inadequate training, and social discomfort [16]. Obviously, students are very concerned about providing negative feedback to their peers, the quality of their feedback, and the consequences of this negative feedback on their peers’ progession [11, 17]. Fortunately, students find that using a strucutred method for providing feedback to peers is useful [10, 11].

### Self-assessment and reflection on performance

Feedback not only has the purpose of improving a learners’ performance, it also acts as a tool to cultivative self-assessment and reflection on performance. Evidence suggests that self-assessment is inaccurate; high performers underestimate themselves, while poor performers overestimate [1, 9]. Receiving external feedback, however, gives learners the opportunity to benchmark their own self assessment against appropriate criteria.

### Effective feedback

Effective feedback is an essential part of the learning process. Effective and regular feedback reinforces good practice, promotes self-reflection, and motivates the learner to work towards their desired outcome [2]. The style of feedback delivery can influence the outcome on the student. Feedback can inspire the student to reflect and improve their performance, or it can be negative and demoralising. We have found that using a structured method, such as Pendleton’s model (1984), illustrated in Table 1, is useful for providing feedback [11, 18–20]. This model of feedback offers learners the opportunity to evaluate their own practice, and identify ways of improving. It also allows for immediate feedback from the observer.

**Table 1:** Feedback model (data from Pendleton et al., 1984) [18]

1. Ask the learner what went well	
2. Tell the learner what went well	
3. Ask the learner what could be improved	
4. Tell the learner what could be improved	

Table 2 provides an activity that allows practice and reflection on the use of Pendleton’s model of feedback.

**Table 2:** An activity: giving and receiving feedback

Activity 1	
Find a colleague who you may be able to practice giving feedback with, using Pendleton’s model of feedback. Note that although the model is simple, it is not easy to adhere to the set framework.	
What were the positive aspects of the way feedback was given?	
What could be improved?	

### Giving effective feedback

Direct observation, and clear goals are needed in the provision of effective feedback, with good performance being reinfoced, and poor performance being corrected [21]. Although provision of constructive feedback detailing both positive and negative aspects of the learner’s performance can be time consuming and difficult, not giving feedback can have a substantial negative effect. If not relayed carefully, feedback can result in a deterioration in performance [4, 21]. If handled poorly, feedback can also cause defensiveness and embarrassment to the learner. Feedback must be non-judgemental and descriptive in nature [22]. There are a number of key principles to consider when conducting effective feedback [2]. Namely, feedback should be:

1. Planned, considering the place, timing and environment
2. Explicit
3. Descriptive
4. Focused on behaviour, not personality
5. Specific
6. Concise
7. Verified by the recipient
8. Honest

The success of a feedback session is dependent on three broad areas: **structure, format, and content**, as outlined below and summarised in Table 3 [2, 23].

**Table 3:** Three key areas of a successful feedback session [2]

**Structure**	
• Schedule the feedback session at convenient time for teacher and student	
• Make the purpose of meeting clear	
• Seating arrangement in the room should show the teacher as a ‘participant’ e.g. round table	
• Feedback should focus on observed knowledge, attitudes and behaviours	
• The format of the session should include self-assessment, teacher assessment and joint development of an action plan	
**Format**	
• The aim of the feedback session is to improve student performance - make this clear	
• Session structure should be made clear - student self-assessment, teacher assessment, joint development of an action plan	
• Use an appropriate feedback model e.g. Pendleton’s positive critique method	
• It is important to both give positive feedback and areas requiring improvement	
• The assessor should provide examples and strategies for improvement	
**Content**	
• Teachers and students need time to prepare respective content for the session	
• The learner should assess their own learning objectives for the clinical placement, including formal objectives and personal objectives	
• The teacher should prepare for the session by making direct observations of the student’s performance, and gaining feedback from others on the team	
• The teacher should review notes and only select a few points to cover	

#### Structure

The timing of feedback needs to be considered for both parties, allowing adequate time for preparation. It may be necessary to ensure the feedback is given in a confidential location, with the purpose of the meeting being made clear to all. The room setting should also be considered, so to not intimidate the student. It is important that feedback is focused on the attitudes, behaviour and knowledge observed, with the use of descriptive words to assist in the understanding of the feedback. Mutual trust and respect should be established, with the shared goal being working towards improving the learner’s performance [9].

#### Format

It is essential the feedback provided is accurate and valuable, with both negative and positive points being made [9]. The aim of the session is to improve the performance of the learner. The steps in the meeting include the learner’s self-assessment, the teacher’s assessment, as well as providing an action plan for future improvement of performance. The key to Pendleton’s model of feedback is to encourage self-reflection and have the student lead the approach to feedback (see Table 1) [18].

#### Content

Adequate time needs to be provided in order for the teacher and learner to prepare for the meeting [9]. Formal learning objectives and personal objectives need to be considered when assessing what learning has taken place. Having the teacher directly observe the student’s performance will provide specific examples of good performance, and areas for improvement. Only a limited number of specific areas for improvement (say two or three of the most crucial only) should be addressed in a single feedback session.

### The role of curriculum design in promoting feedback

The curriculum should be deliberately designed to inspire students to engage in feedback [3]. Feedback should be viewed as a required element of any curriculum, and central to student learning. Interventions to promote feedback need to ‘permeate’ the curriculum and the culture of organisations, to ensure learners are able to identify appropriate standards to apply to their work [3]. Fruitful learning environments should be constructed by students to practice and actively build on their ability to make judgements about their own work. Comparisons of performance should be encouraged early in the curriculum. This helps students to develop an awareness of their current capabilities, and plan for their own learning needs.

Self regulated learning (SRL) offers a process that empowers students to actively engage in and direct their own learning [24]. The use of SRL helps students to set goals, actively engage in learning activities, and monitor their own progress and actions in achievement of their goals [24, 25]. Feedback can be given to students on their use of SRL to encourage strategies in learning that are clear and specific, self-monitored, and reflected upon [25]. The challenge for educators is to systematically build self-analysis as an expectation within the curriculum. Regular self-analysis helps to build habits that promote comparison between self-analysis and external analysis [3]. Tips for designing a curriculum that positions feedback as a key attribute include:

- Orientate the students to the purpose of feedback
- Orientate students to methods of feedback
- Promote opportunities for multiple tasks with formative assessment and feedback
- Develop incremental challenges for tasks
- Provide opportunities for students to not only receive, but practice giving feedback1

## Conclusion

Feedback is an essential component of the learning process, and is considered an integral part of the curriculum. Despite the growing body of literature surrounding feedback, there is little agreement on the best approach. No single feedback model will work across all clinical contexts. Each clinical educator needs to engage in the process of feedback, and can take the opportunity to develop their own best practice. Regular and effective feedback helps to reinforce good practice and motivate the learner towards the desired outcome. Because skills in giving and receiving feedback are rarely taught to health professional students, they are often lacking in clinicians. Direct observation and feedback offers a powerful tool to inform the learner of their progress at a specific point in time [24, 26]. In order to increase the efficacy of the educational process, it is important for both learners and teachers to understand the purpose and structure of feedback.

### Take-home message

**Table:** 

• The learning environment should foster feedback.	
• Effective feedback has the potential to improve skills and change the learner’s behaviour.	
• Using a structured format to provide feedback (such as Pendleton’s model), assists in self-reflection and the provision of clear, constructive advice.	
• The curriculum should be deliberately designed to inspire students to engage in feedback.	

## Data Availability

Not applicable.

## Abbreviation

SRL: Self regulated learning

## References

[1] Van den Berg I, Admiraal W, Pilot A (2006). Peer assessment in university teaching: evaluating seven course designs. Assess Eval High Educ.

[2] Burgess A, Mellis C (2015). Feedback and assessment during clinical placements: achieving the right balance. Adv Med Educ Pract.

[3] Boud D, Molloy E (2013). Rethinking models of feedback for learning: the challenge of design. Assess Eval High Educ.

[4] Zahid A, Hong J, Young C (2017). Surgical supervisor feedback affects performance: a blinded randomized study. Cureus.

[5] Shepard LA (2000). The role of assessment in a learning culture. Educ Res.

[6] Hattie J, Timperley H (2007). The power of feedback. Rev Educ Res.

[7] Branch WT, Paranjape A (2002). Feedback and reflection: teaching methods for clinical settings. Acad Med.

[8] Ende J (1983). Feedback in clinical medical education. Med Educ.

[9] Davis DA, Mazmanian PE, Fordis M, Van Harrison R, Thorpe KE, Perrier L (2006). Accuracy of physician self-assessment compared with observed measures of competence: a systematic review. JAMA.

[10] Telio S, Ajjawi R, Regehr G (2015). The “educational alliance” as a framework for reconceptualizing feedback in medical education. Acad Med.

[11] Burgess A, Roberts C, Black K, Mellis C (2013). Senior medical student perceived ability and experience in giving peer feedback in formative long cases examinations. BMC Med Educ.

[12] Burgess A, Clark T, Chapman R, Mellis C (2012). Senior medical students as peer examiners in an OSCE. Med Teach.

[13] Topping KJ (2005). Trends in peer learning. Educ Psychol.

[14] Burgess A, McGregor D, Mellis C (2014). A systematic review of peer assisted learning (PAL) in medical schools. BMC Med Educ.

[15] Burgess A, Roberts C, Black K, Mellis K (2014). Student ability to assess their peers in long case clinical examination. IJOCS.

[16] Cassidy S (2006). Developing employability skills: peer assessment in higher education. Educ Train.

[17] Falchikov N, Goldfinch J (2000). Student peer assessment in higher education a meta-analysis comparing peer and teacher marks. Rev Educ Res.

[18] Pendleton D, Schofield T, Tate P, Havelock P (1984). The consultation: an approach to learning and teaching.

[19] Burgess A, van Diggele C, Mellis C (2018). Faculty development for junior health professionals. Clin Teach.

[20] Burgess A, Roberts C, van Diggele V, Mellis C (2017). Peer teacher training program: interprofessional and flipped learning. BMC Med Educ.

[21] Cantillon P, Sargeant J (2008). Giving feedback in clinical settings. BMJ.

[22] Chowdhury R, Kalu G (2004). Learning to give feedback in medical education. Obstet Gynaecol.

[23] Bienstock JL, Katz NT, Cox SM, Hueppchen N, Erickson S (2007). To the point: medical education reviews – providing feedback. Am J Obstet Gynaecol.

[24] Zimmerman BJ (2002). Becoming a self-regulated learner: an overview. Theory Pract.

[25] Leggett H, Sanders J, Roberts T (2017). Twelve tips on how to provide self-regulated learning (SRL) enhanced feedback on clinical performance. Med Teach.

[26] Huggett N, Jeffries WB. An introduction to medical teaching: Springer Netherlands; 2014. 10.1007/978-94-017-9066-6.

